# Community resilience to climate change and non-communicable disease vulnerability in Yaoundé, Cameroon: insights from community-based systems dynamics

**DOI:** 10.3389/fpubh.2025.1718345

**Published:** 2025-12-19

**Authors:** Felix Assah, Blanche Nfondoh, Edwin Ngwa, Tchouaffi Awah Kum, Boris Elouna, Yves Wasnyo, Lidia Maria de Oliveira Morais, Meelan Thondoo, Feyisayo Wayas, Gladys Odhiambo, Janel Phillips, Nina Abrahams, Tolullah Oni, Estelle Victoria Lambert, Waleska Teixeira Caiaffa, Leandro Garcia, Georgiana Gordon-Strachan, Lambed Tatah

**Affiliations:** 1 Health of Population in Transition Research Group, University of Yaoundé 1, Yaoundé, Cameroon; 2 Observatory for Urban Health in Belo Horizonte, Federal University of Minas Gerais, Belo Horizonte, Brazil; 3 MRC Epidemiology Unit, University of Cambridge, Cambridge, United Kingdom; 4 Research Centre for Health through Physical Activity, Lifestyle and Sport (HPALS), University of Cape Town, Cape Town, South Africa; 5 Center for Global Health Research, Kenya Medical Research Institute, Kisumu, Kenya; 6 Tropical Metabolism Research Unit, University of the West Indies, Kingston, Jamaica; 7 Centre for Public Health, Queen's University Belfast, Belfast, United Kingdom

**Keywords:** community-based system dynamics, global south, climate change, climate resilience, complex systems, intersectorality, urban health

## Abstract

**Background:**

Climate change and rapid urbanisation have intensified flood risk in Global South cities, exacerbating health inequities, especially through non-communicable diseases (NCDs). However, little is known about how community resilience strategies to key climate change consequences like flooding affect NCD risk in rapidly growing cities of the Global South.

**Methods:**

We used a Community-Based System Dynamics (CBSD) approach to examine flood resilience strategies, the determinants, and health implications of these strategies in Yaoundé, Cameroon. The study included semi-structured interviews and a participatory modelling workshop with 12 purposively sampled community stakeholders (including from the municipality, urban planning, civil society organisation, local leadership, and people affected by flooding), accompanied by an iterative development and analysis of a causal loop diagram (CLD) to capture key variables, relationships, and feedback loops.

**Findings:**

The finalised CLD incorporated 14 key variables and featured five major feedback loops (four reinforcing, one balancing) that shape flood resilience. Community-led strategies—such as waste management, tree planting, drainage maintenance, and the construction of flood-resistant infrastructure—were driven by municipal support, enforcement of planning rules, and adaptation within informal settlements. Participants described how these strategies improved hygiene, enhanced access to food and physical activity spaces, and reduced immediate health risks. However, political interests and inadequate enforcement constrained long-term resilience. Importantly, the study identified plausible pathways through which community actors perceived flood resilience strategies influenced diet and physical activity, the main NCD risk factors, thus highlighting the climate change-NCD syndemic in an urban African context.

**Conclusion:**

Participatory CBSD provided novel, systems-level insights into community resilience, revealing dynamic feedback between local action, governance, and health. Integrating community-led approaches into formal disaster risk management and urban health policy is essential for sustainable, equitable resilience.

## Introduction

Climate change continues to drive up the frequency and intensity of extreme weather events, particularly flooding, which affects over 1.8 billion people and causes 10 of 1,000 of deaths worldwide annually (1, 2). Floods not only cause direct injuries and fatalities but also exacerbate outbreaks of infectious and vector-borne diseases, increase food and water insecurity, and disrupt mobility, public spaces and health services. The health risks are not evenly distributed: rapidly urbanising Global South African cities are disproportionately affected, primarily because of the convergence of hazards such as urban sprawl, large informal settlements, infrastructure deficits, weak governance, and climate and other environmental and social stressors (3, 4). These hazards interact synergistically with non-communicable disease (NCD) risk factors and exacerbate health and socioeconomic disparities (3, 5), hence the climate change—NCD syndemics.

Yaoundé, the capital city of Cameroon, provides a striking example of the urban challenges associated with frequent and devastating floods. The city, characterised by rapid population growth and burgeoning informal settlements, experiences severe recurrent flood-related disasters, driven by a combination of heavy rainfall (2,000 mm per year), rapid urban expansion, poor drainage infrastructure, and insufficient disaster risk governance (6, 7). The country’s National Climate Change Adaptation plan, as a whole, has yet to account for the effects of climate change (8). Prior to the implementation of the African Development Bank’s sanitation projects (Yaoundé sanitation project (PADY)-2005 and Yaoundé City Sustainable Enhanced Drainage and Sanitation Project (PCADY)-2021), the lack of rainwater drainage resulted in systematic flooding in the rainy season (9). Floods have reduced with the implementation of PADY 2005 and 2021 projects, but still result in substantial damage to housing, livelihoods, public health, and critical urban services (10).

Traditional top-down disaster management frameworks have frequently failed to adequately account for local contexts and community capacities, limiting their effectiveness in fostering sustainable resilience (11). Addressing complex disaster vulnerabilities, therefore, necessitates integrated and inclusive approaches that highlight community efforts. Community resilience strategies (including mitigation, adaptation, coping, and bounce-back strategies), the drivers and outcomes of these strategies often interact in non-linear and complex ways that make it challenging to know which interventions are ultimately beneficial. A shared understanding of the complex interactions of these factors is key to building community resilience around climate change, as community-driven resilience strategies rely on effective intersectoral collaboration between governmental, non-governmental, and community actors. Such collaboration favours resilience strategies that are context-specific, culturally appropriate, and sustainable (12). In Yaoundé, examples of community strategies include community-driven early warning systems, local flood risk education, and collective actions to improve drainage and waste management systems (13), underscoring the value of participatory governance and local resource mobilisation (11).

Community-Based System Dynamics (CBSD), a participatory systems-thinking approach, provides a robust methodological framework for understanding the intricacies and interdependencies within flood resilience systems from the perspective of affected communities, and unveiling a tapestry of complex feedback interactions (14). CBSD involves community stakeholders in the co-creation of conceptual models, typically visualised through causal loop diagrams, representing key variables, relationships, and feedback loops. It can be applied to facilitate shared learning on the local dynamics of flood risk and community resilience strategies (11, 14, 15). This participatory modelling approach has demonstrated effectiveness in various contexts, including Limbe, Cameroon, and Belo Horizonte, Brazil, by revealing hidden vulnerabilities, enhancing stakeholders’ shared understanding, and identifying critical leverage points to inform more effective policy and infrastructure intervention in the system in focus (11, 16).

Participatory systems thinking approaches for climate resilience are growing in health research, but major knowledge gaps remain. Their use in the Global South is limited, restricting our understanding of how to systematically integrate community strategies into disaster risk reduction frameworks across diverse urban contexts. Furthermore, little is known about how community flood resilience efforts affect urban health, particularly NCDs, through changes in leading behavioural risk factors like diet and physical activity, a relationship increasingly described as the climate change-NCD syndemic (17, 18).

This study adds to a growing body of literature demonstrating the utility of participatory systems thinking approaches in climate adaptation planning. It aims to address the knowledge and action gaps by exploring community resilience strategies to flooding in Yaoundé. Specifically, the objectives are to analyse community resilience strategies, investigate complex feedback interactions among determinants, strategies, and health-related impacts, and explore how community actors perceive these strategies’ potential influence on NCD risk factors such as diet and physical activity. By doing so, this research seeks to contribute valuable insights into effective participatory frameworks for climate resilience, relevant not only to Cameroon but also transferable to rapidly urbanising cities across the Global South. The study also aims to inform the design of inclusive urban policies that address both climate adaptation and health equity. This study adds to a growing body of literature demonstrating the utility of participatory systems approaches in climate adaptation planning. Accordingly, this study addresses the following research question: “How do community actors in Yaoundé perceive and enact strategies to build resilience to urban flooding, and through what interacting drivers and consequences might these strategies influence health-related determinants and equity?”

## Method

### Study design

We used a CBSD approach to investigate community resilience strategies to flooding in Yaoundé, as part of the Global Diet and Activity Research (GDAR) network’s multi-site research, co-developed and implemented in the Cameroonian context (16). This work package focused on unpacking urban community action for climate and NCDs in Belo Horizonte (Brazil), Kisumu (Kenya), Kingston (Jamaica), and Yaoundé (Cameroon) (16). The GDAR Network is a transdisciplinary global health partnership established in 2017 to address the upstream drivers of NCD, such as poor diets and physical inactivity, particularly in urban settings of low- and middle-income countries in the Global South. It focuses on generating policy-relevant research and strengthening local capacity by studying how physical, policy, and social environments influence health behaviours (19).

CBSD was chosen due to its capacity to capture the complex, adaptive nature of urban flood resilience, surface local knowledge, and build shared understanding and consensus among diverse community stakeholders in contexts where formal data systems may be weak or fragmented. Our CBSD process comprised four core phases: (1) semi-structured interviews to elicit individual mental models of flood risk and resilience; (2) thematic analysis; (3) a participatory modelling workshop to co-develop a causal loop diagram (CLD) draft; and (4) finalisation of the CLD through iterative stakeholder feedback. This phased approach facilitated the building of shared understanding and depiction of system features that drive community resilience.

### Team training and instruments

The research team comprised researchers from the University of Yaoundé and GDAR partner institutions. The team underwent a 3-day CBSD training, led by an expert (LG), utilising the resources and session outlines available in Hoveman’s CBSD guidance (14). The training covered the use of the interview guide, virtual collaborative platforms (Mural) for real-time mapping and Kumu for CLD visualization, alongside cross-site webinars with Belo Horizonte, Kingston, and Kisumu teams to harmonise methodological approaches and troubleshoot platform-specific challenges (16).

The interview guide was structured per CBSD best practices (14), beginning with broad questions on flood experiences and progressing to targeted probes on causal factors, causal interdependencies, leverage points, and adaptation strategies (16). Visual mapping templates provided in Mural structured participants’ contributions, which allowed the elicitation of individual mental models through three steps: listing key variables, ranking their importance, and sketching directional links. Kumu was used to combine the individual mental models into a CLD, prune the CLD, and analyse its emerging properties (16).

### Participant identification and recruitment

We used purposive and snowball sampling to recruit 12 community actors with diverse perspectives on flooding and climate change. Initial community actors were identified through local advocacy groups and municipal flood committees, including civil society leaders, neighbourhood association heads, local government representatives, faith-based organisation coordinators, and environmental NGOs known to the research team. Each respondent nominated additional participants until thematic saturation was reached (20), ensuring the inclusion of both male and female voices across socioeconomic strata. To minimise selection bias, we explicitly asked initial contacts to nominate people beyond their usual collaborators, including individuals who had been critical of, or less engaged in, existing flood initiatives and from neighbourhoods most frequently affected by flooding. Some of these community actors had been engaged in previous GDAR work packages.

### Data collection and CLD development

In-person semi-structured interviews were conducted in French or English, at the interviewee’s preference, by bilingual field facilitators during the 2024 rainy season (May–July). Data collectors were paired, one captured the data and built a mental model on the Mural platform in real-time and the other interviewed the participants. Interview sessions lasted between 45 and 60 min and followed an interview guide developed by the GDAR team and described in Morais et al. (16). Participants were allowed to revise their mental models at the end of the interviews. Interview transcripts and individual mental models were coded thematically in Excel using an *a priori* codebook derived from GDAR’s cross-site analysis to capture themes related to flood resilient strategies, as well as the determinants and outcomes of these strategies (see supplementary material in Morais et al. (16)).

We used a structured procedure to translate the themed qualitative data into CLD variables and links. Texts that referred to a specific strategy, resource, behaviour or outcome were first listed as candidate variables, and associated statements of influence (for example, “X increases Y”) were recorded as candidate links. Variables that described the same underlying construct, were used interchangeably by participants, or consistently had the same direction of effect were aggregated into a single variable, with labels chosen to remain close to participants’ wording but understandable to a broader audience.

The coded variables were used to replicate one of the mental models as the starting CLD on Kumu. Additional variables and linkages from the rest of the individual mental models were used to revise and update the CLD successively. During iterative reviews of the diagram, the research team examined the emerging CLD to remove elements that added complexity without altering the main dynamics and to ensure that each retained variable and link could be traced back to at least one transcript excerpt or mental model, with its polarity and direction consistent with the underlying accounts. Where a proposed link was not clearly supported by the data, we either removed it or retained it only when it was also supported by prior empirical or theoretical work on flooding, resilience and health. This preliminary CLD was therefore considered a data-grounded, yet provisional, representation of participants’ mental models. This draft CLD was used as the working template for a 1-day participatory workshop attended by the 12 interviewed stakeholders, who were organised into three groups to refine and merge their CLDs into one agreed CLD. Each group reviewed specific sections of the diagram, suggested additions and deletions, and proposed changes in causal direction where they felt the initial representation did not reflect their experience; points of disagreement were discussed in plenary until a shared wording or structure was reached, or, where this was not possible, links were explicitly marked as context-dependent. Facilitators from the research team documented emergent group discussions and consensus points.

The CLD resulting from the participatory workshop remained complex and challenging to follow. Further analysis by the research team resulted in pruning of redundant or intermediate elements and links that did not significantly contribute to the key dynamics captured in the CLD, while highlighting the emergent feedback loops. The prioritisation of the retained feedback loops was determined by the number of nodes and the narratives from the group discussions. As a final consistency check, the research team re-examined the agreed CLD against the coded transcripts and mental models to ensure that all retained variables and links were traceable to at least one data source and remained consistent with participants’ accounts. To annotate the structure of the CLD, we identified feedback loops as either balancing (B) or reinforcing (R). Reinforcing loops were characterised by changes in variables that amplify through the loop, leading to either growth or decline. In contrast, balancing loops show a tendency to stabilise the system by counteracting changes. This classification provided insight into the dynamic behaviour of the system represented in the CLD.

## Results

**Figure 1:**
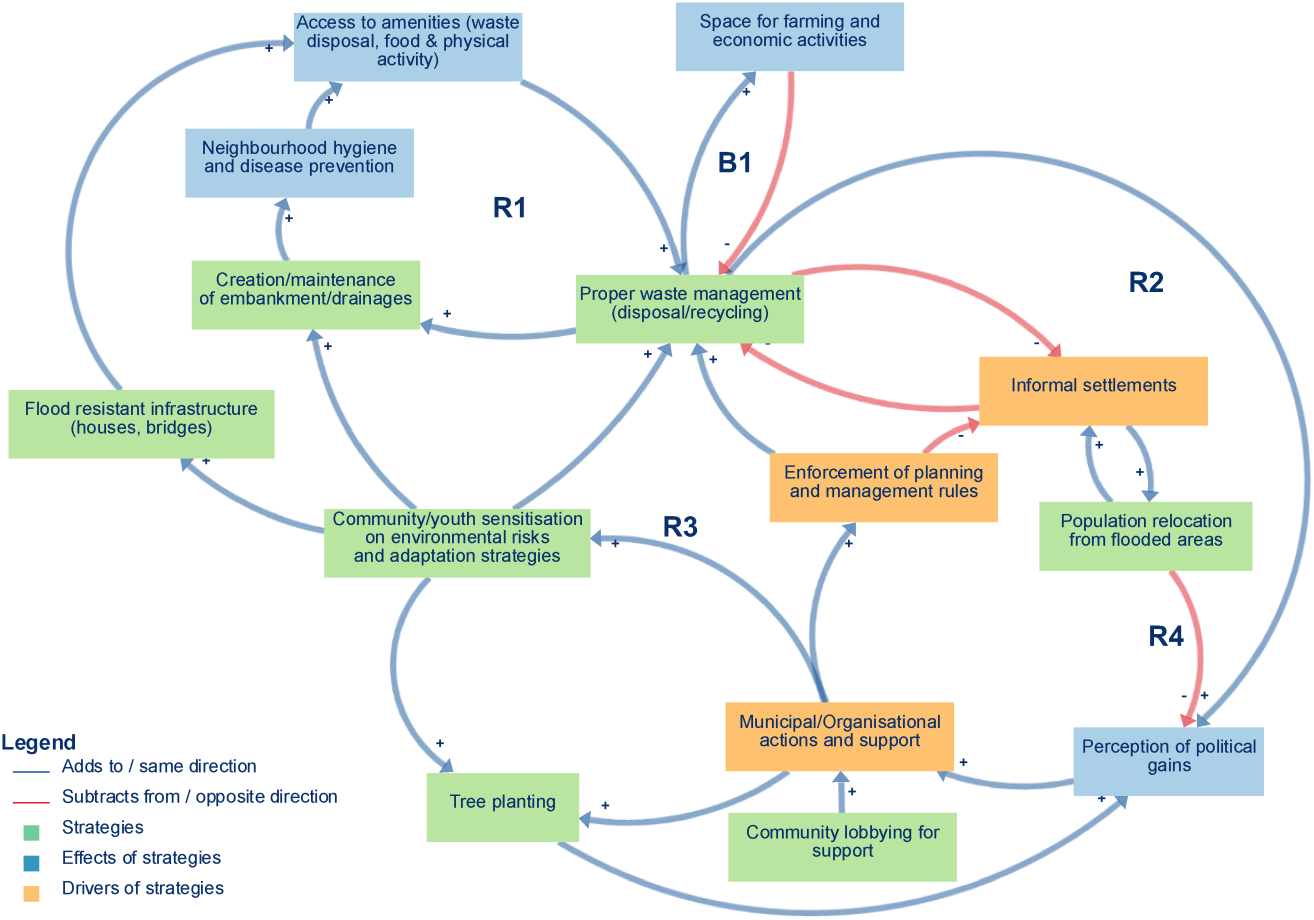
Causal loop diagram of flood-related community resilience strategies, their drivers and effects, organised into three themes (coloured boxes), and five key feedback loops in Yaoundé, Cameroon.

**Figure A1:**
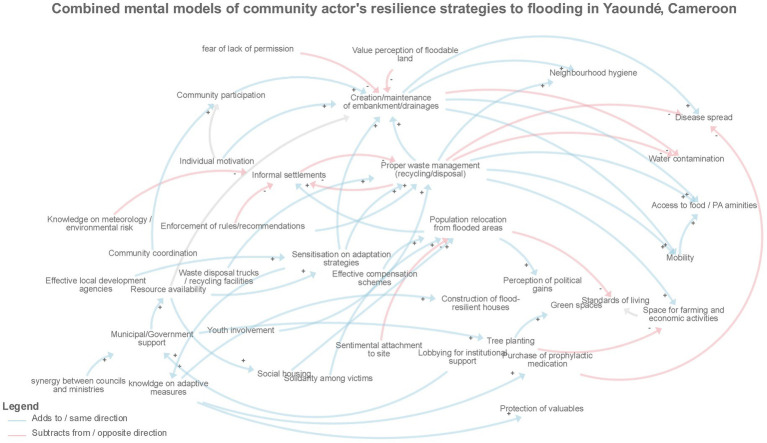
Combined metal model of community actors’ flood resilience strategies, their drivers and effects, in Yaoundé, Cameroon.

The resulting CLD (Figure 1) illustrates the shared understanding of community resilience strategies employed in response to flooding in Yaoundé, Cameroon. The CLD represents a pruned combined mental model (Figure A1). It comprises 14 variables organised into three main categories—seven strategies, three drivers of strategies, and four effects of strategies—interconnected by 16 positive causal relationships (indicating variables that change in the same direction) and five negative causal relationships (indicating variables that change in opposite directions). Five key feedback loops, four reinforcing (R1, R2, R3, R4) and one balancing (B1), were identified and highlighted in the diagram. The CLD integrates relationships that participants explicitly articulated during interviews and the workshop with additional linkages and feedback loops that were inferred by the research team when consolidating individual mental models. In the descriptions below, we indicate where links and loops were directly described by participants and where they reflect this analytical synthesis. The variables, categories, relationships, and feedback loops are described below.

### Strategies (green boxes)

The theme coded as *strategies* encompasses various community-led initiatives aimed at reducing flood impacts and enhancing resilience. These strategies focus on mitigation, adaptation, coping, and resilience measures aimed at safeguarding communities from the adverse effects of floods (21, 22). Key strategies included *proper waste management (disposal and recycling), creation and maintenance of embankments and drainages, community and youth sensitisation on environmental risks and adaptation strategies, tree planting, building flood-resistant infrastructure, population relocation from flooded areas,* and *community lobbying for support*. Participants named each of these concrete actions, while the grouping under the overarching category of “strategies” and their organisation in Figure 1 were developed by the authors to summarise these accounts.

Participants emphasised the importance of *proper waste management (disposal and recycling)* as a crucial measure for maintaining functional drainage systems and reducing flood risks. The accumulation of solid waste, especially plastic debris, obstructs natural and artificial drainage systems, leading to water stagnation and urban flooding. Proper waste management activities included encouraging waste segregation, community recycling programs, and public awareness campaigns on waste disposal. Additional named activities included the establishment of designated waste disposal sites, periodic clean-up exercises, and collaboration with waste management agencies. These links between waste practices, drainage and flood risk were explicitly described by participants.

Similarly, the *creation and maintenance of embankments and drainage* systems to provide dedicated pathways for excess rainwater are considered an effective strategy for managing flooding. While municipalities have built some drainage systems, their upkeep is largely carried out by communities during organised clean-up days. In many cases, communities themselves create additional drainage channels and reinforce embankments using sandbags.

*Community and youth sensitisation on environmental risks and adaptation strategies* is recognised as a key approach to strengthening resilience. Awareness campaigns and educational activities help residents, especially younger generations, understand local hazards and the actions they can take to reduce their vulnerability. Through workshops, school programs, and neighbourhood initiatives, communities are encouraged to adopt safer practices and to actively participate in collective adaptation efforts.

*Tree planting* emerged as a fundamental action to enhance soil stability by preventing soil erosion, regulating water absorption, reducing surface runoff, and mitigating flooding. The planting of native species that thrive in flood-prone areas ensures long-term sustainability and ecological balance. Additionally, reforestation projects and green buffer zones around flood-sensitive locations can significantly lessen the intensity of floodwaters. This activity also scores some political points for the actors. The interpretation of tree planting as both an environmental and political strategy reflects our synthesis of several participants’ accounts.

*Building flood-resistant infrastructure,* such as reinforced houses, elevated foundations, and robust bridges, was identified as critical for minimizing damage and strengthening community resilience. Participants highlighted the use of durable materials, extended foundations beyond flood beds, and systems for suspending furniture during floods. Low-cost wooden bridges were often constructed to restore mobility after flooding. These measures help ensure that homes, businesses, and transport networks remain functional and safe during flood events.

*Population relocation from flooded areas* is sometimes used, but it presents both logistical challenges and socioeconomic implications for affected communities. *Community lobbying for support* played a crucial role in mobilising resources and engaging additional stakeholders.

### Drivers of strategies (orange boxes)

Key drivers (i.e., positive factors that enable or negative factors that inhibit the strategies) that influence the implementation of the named resilience strategies included *municipal or organisational support, enforcement of planning and management rules, and informal settlements*. Participants did not usually use the term “drivers” themselves; this category and the specific links shown in Figure 1 represent our synthesis of how they described enabling and constraining conditions.

Increased *municipal or organisational actions and support* significantly enhance the community’s ability to implement strategies related to infrastructure improvement and environmental management practices through technical assistance, funding, policy frameworks, and infrastructure development. *Municipal or organisational action and support* often extend to early warning systems, climate adaptation projects, and urban planning regulations that prevent settlement in high-risk flood zones. However, the consistency, accessibility, and political will behind these actions vary, influencing how effectively resilience strategies are executed.

*Informal settlements* were acknowledged as both drivers and constraints, representing areas requiring urgent resilience actions but often lacking formal support. Communities in unregulated, high-risk zones experience higher exposure to environmental hazards (flooding, landslides, poor sanitation), limited access to municipal services and disaster mitigation infrastructure, and weak enforcement of zoning laws and urban planning regulations. Despite these challenges, *informal settlements* drive resilience strategies by fostering community-led adaptation solutions, including grassroots mobilisation to implement local flood prevention methods, informal networks that support risk-sharing and mutual aid in times of crisis, and innovation in housing and drainage modifications to withstand environmental pressures. However, due to the lack of formal recognition, these communities often face barriers to securing government funding, disaster relief, and long-term planning. Addressing resilience within informal settlements requires integrated urban policies, collaborative planning, and capacity-building initiatives to bridge the gap between municipal support and community-led adaptation. These nuanced roles of informal settlements emerged from participants’ narratives, while their representation as a single node connecting multiple strategies in the CLD is an analytical consolidation.

The *enforcement of planning and management rules,* mainly by municipal authorities, includes monitoring land use, ensuring construction adheres to approved plans, and has the potential to curb informal settlements and promote proper waste management. This enforcement also involves overseeing waste management practices, such as requiring appropriate waste collection, disposal, and recycling, as well as conducting regular inspections and community education.

### Effects of strategies (blue boxes)

This refers to the outcomes and consequences of the said strategies. The implementation of resilience strategies has led to notable improvements in various aspects of community well-being, infrastructure, and environmental management. These include foundational gains such as *improved access to amenities—waste disposal, food, and spaces for physical activity—*which collectively enhance *public health* and *neighbourhood hygiene*. Building on this, *the creation of dedicated spaces for farming and economic activities* has strengthened livelihoods and food security. Ultimately, these outcomes contribute to a *perception of political gains*. Participants described these outcomes in relation to their own neighbourhoods, and we grouped them under the broader category of “effects” to capture their shared direction of influence.

Participants noted that enhancing *access to amenities* like waste disposal systems by establishing proper garbage collection and recycling programs has reduced clogged drainage, minimizing flood risks caused by waste blockages, food security through urban farming initiatives and improved land management have facilitated access to fresh produce thereby decreasing reliance on disrupted supply chains during floods, spaces for physical activity in public spaces, parks, and recreational areas have been preserved or repurposed, ensuring communities have places for exercise and social interactions.

*Neighbourhood hygiene* significantly reduced the immediate health risks associated with floods, such as infectious diseases. Participants emphasised that enhanced hygiene measures have significantly improved public health through better sanitation infrastructure, including improved drainage, latrine systems, and proper waste treatment, community-led clean-up initiatives ensuring stagnant water and waste accumulation are swiftly addressed, public health awareness campaigns, and educating residents on flood-related disease prevention. These efforts directly reduce health risks, decreasing the burden on local healthcare systems and promoting cleaner and safer living environments.

Participants also highlighted that the *perception of political gains* can negatively impact resilience-building activities, particularly when political interests overshadow genuine community needs. This includes: short-term decision-making driven by electoral cycles rather than long-term sustainability, allocation of resources based on political alliances rather than objective risk assessments, and selective prioritisation of projects that benefit political figures rather than vulnerable populations. Political interests may influence which resilience measures receive funding and attention, often leading to delays or misallocation of resources. Conversely, in cases where politicians align with community-driven resilience efforts, they can facilitate policy enforcement, budget allocations, and advocacy for environmental reforms. Our interpretation of these dynamics as a distinct “political gains” effect node and its links to other variables in the CLD reflects the consolidation of these various accounts.

### Key feedback loops

Five key feedback loops, four reinforcing (R1, R2, R3, R4) and one balancing (B1), were identified in the analysis. A reinforcing loop amplifies change in a system, leading to growth or decline. In contrast, a balancing loop counteracts change, helping to stabilise the system, thus providing insight into systemic interactions and their implications for resilience-building. These loops illustrate potential leverage points for policy and action, highlighting areas where targeted interventions could significantly enhance resilience outcomes. Participants sometimes described chains of cause and effect, but they rarely framed these explicitly as feedback cycles; the five loops presented below were refined by the authors when overlaying individual narratives onto the combined CLD. Where a feedback loop was not fully articulated by participants as a closed cycle, we present it as a plausible mechanism inferred from the combined CLD and existing literature, rather than as a causal structure that has been empirically demonstrated.

**Figure 2:**
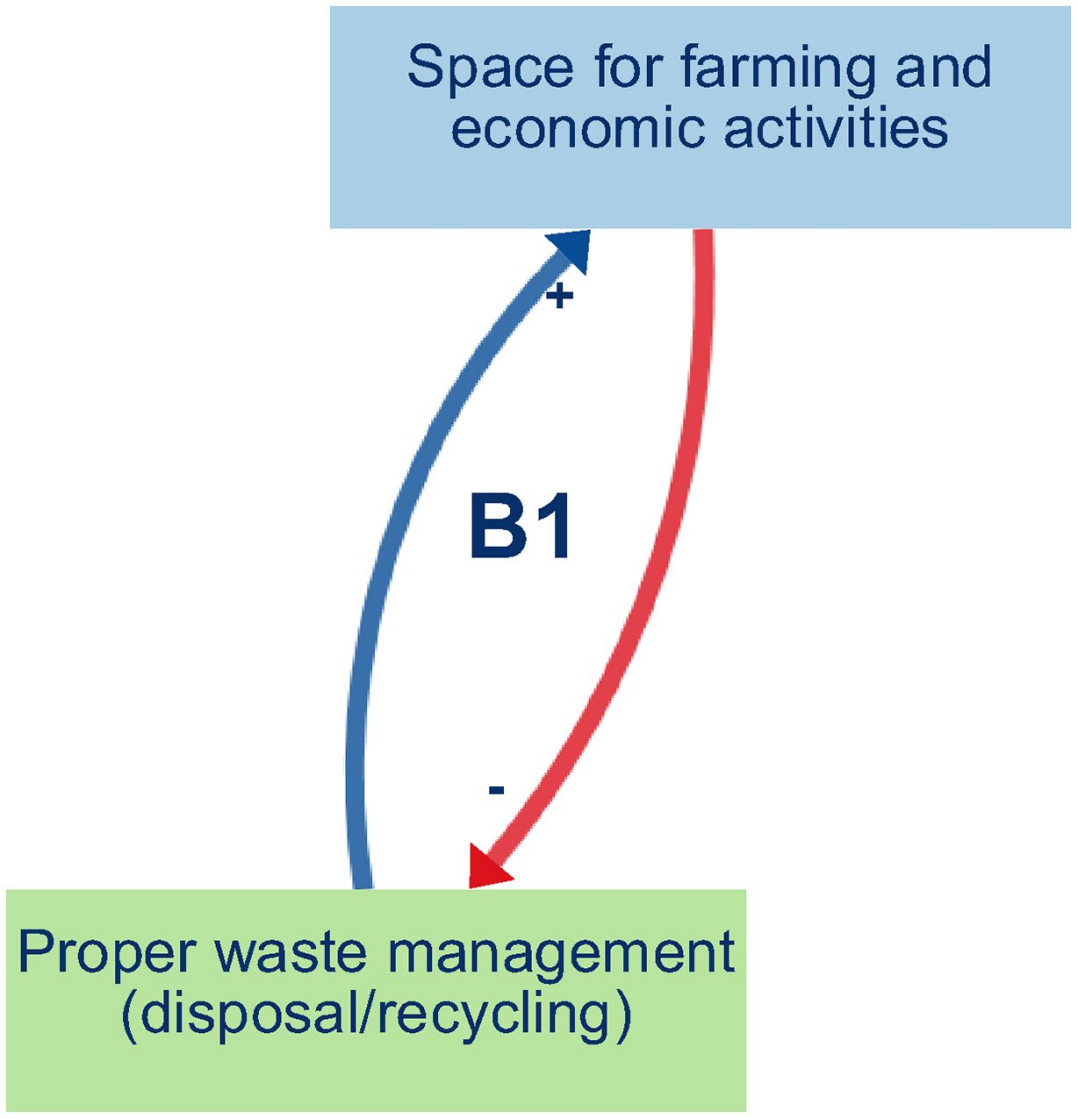
Balancing loop B1.

**Figure 3:**
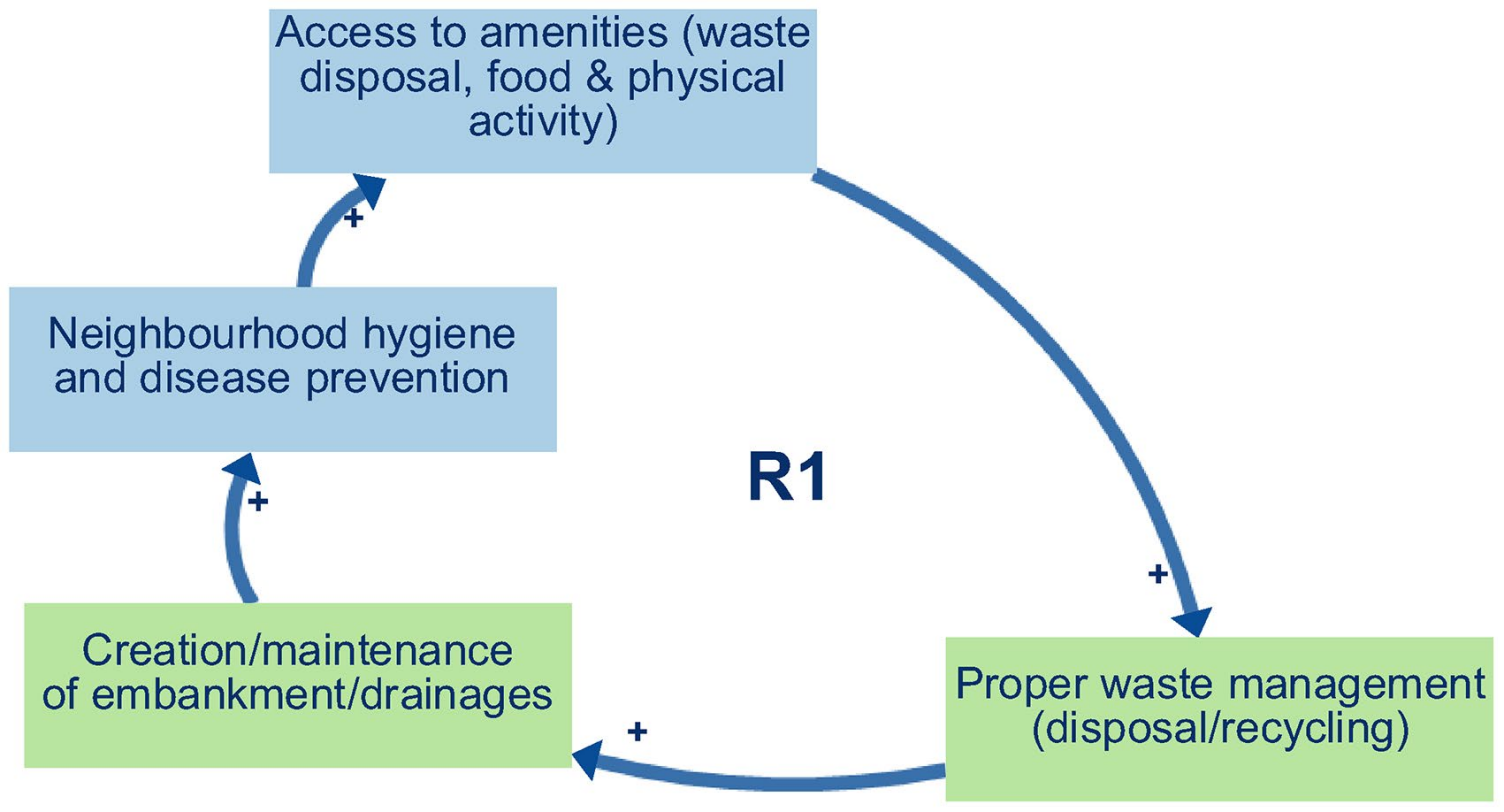
Reinforcing loops R1.

**Figure 4:**
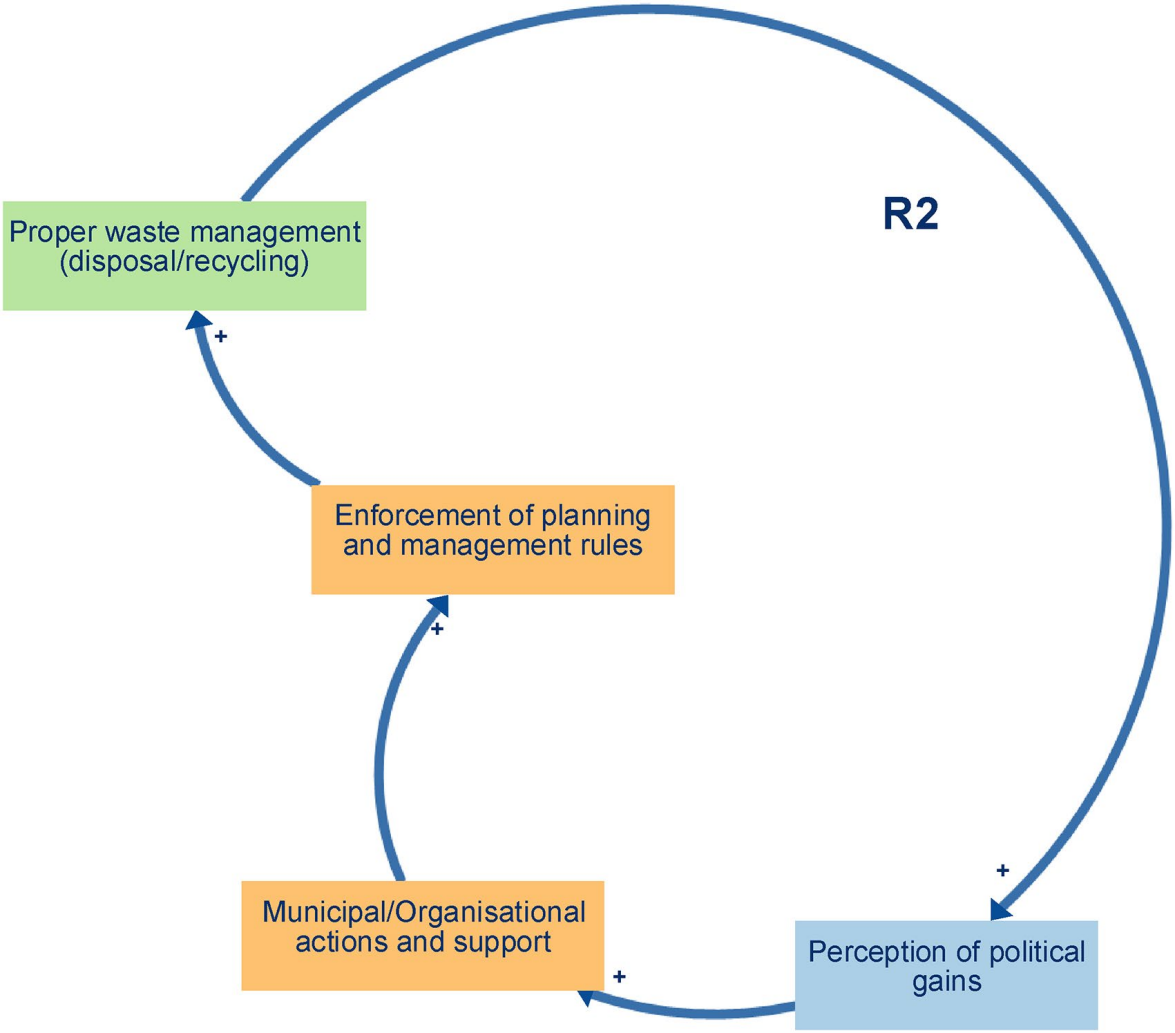
Reinforcing loops R2.

**Figure 5:**
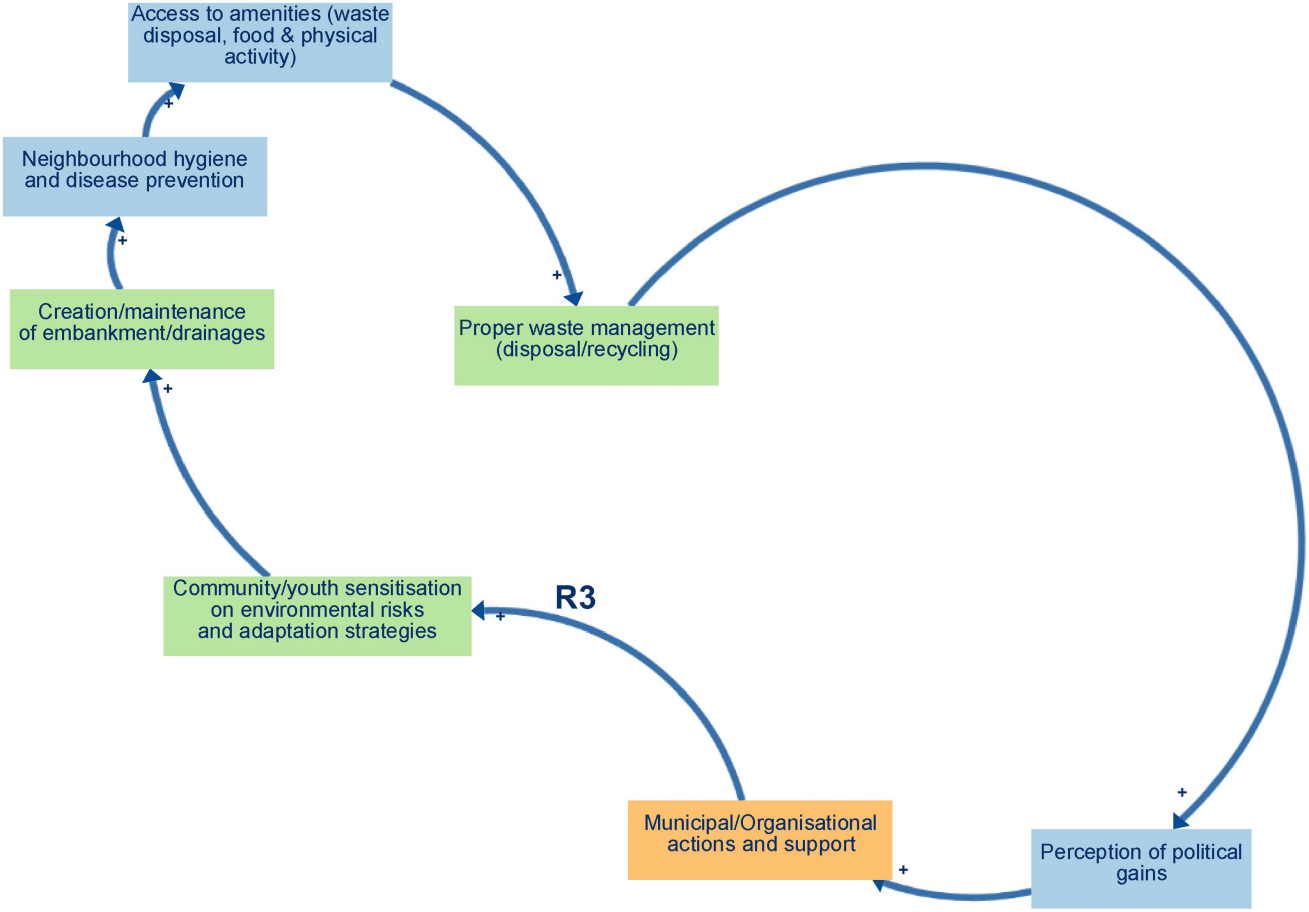
Reinforcing loops R3.

**Figure 6:**
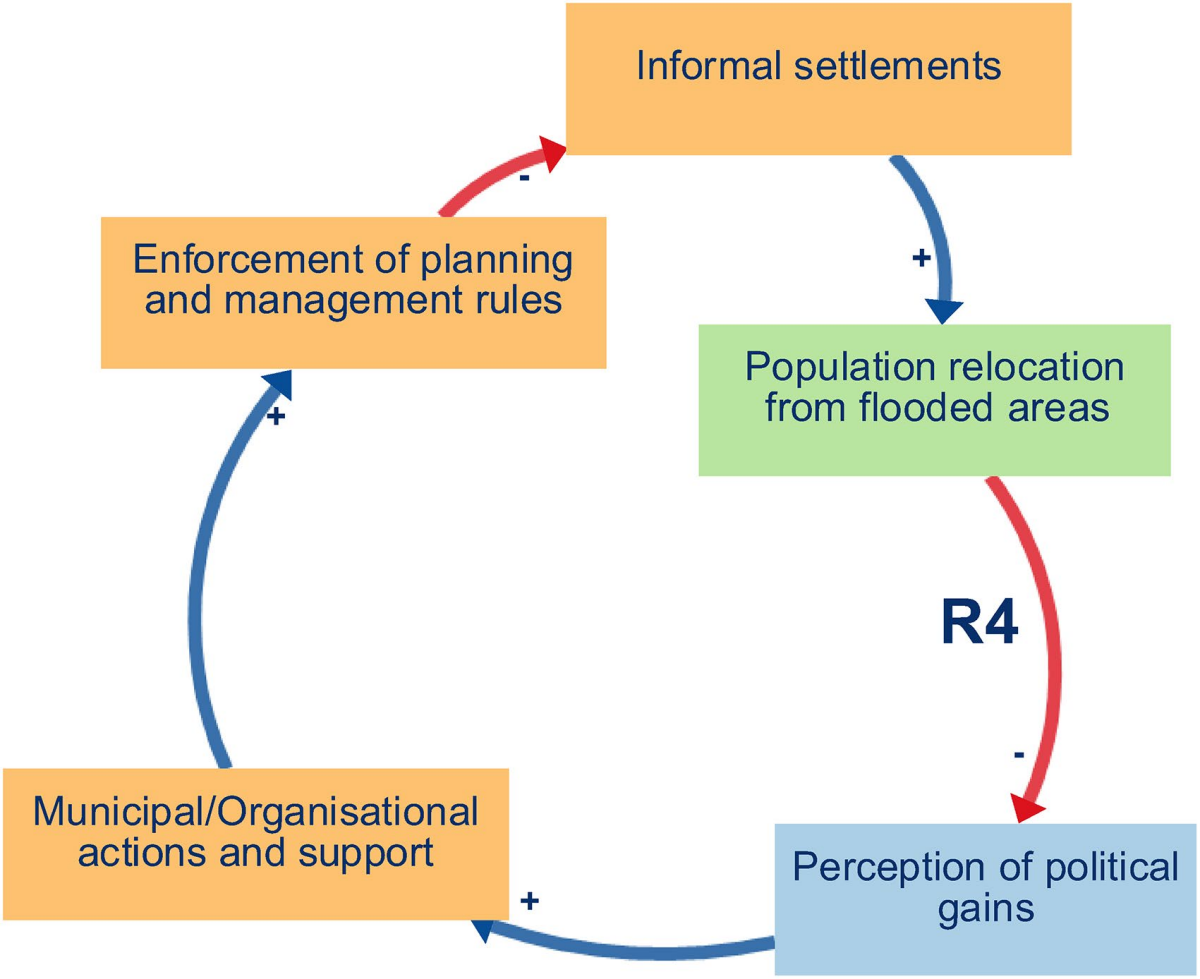
Reinforcing loops R4.

- Balancing loop B1 (Proper waste management and space for farming and economic activities) (Figure 2) captures a self-regulating process in which decreased waste disposal and recycling practices lead to the accumulation of solid waste on vacant lots and peri-urban agricultural fields, clogging drainage channels, and degrading soil quality. This, in turn, shrinks the amount of usable land for urban farming, undermines local markets and household income generation, and limits opportunities for small-scale economic ventures. As financial pressures mount, community members may prioritise short-term relief through informal dumping over long-term land conservation, thereby reinforcing the loop’s balancing effect. Ultimately, unless proper waste management measures are strengthened, the growth of urban agriculture and micro-enterprises will be constrained, compromising food security and economic resilience in Yaoundé’s flood-prone neighbourhoods.
- Reinforcing loop R1 (Environmental Management and Hygiene) (Figure 3) captures a cycle in which effective waste disposal and recycling practices reduce blockages in drains and supports the regular upkeep of embankments and drainage networks. This leads to improved neighbourhood hygiene, a decline in water- and vector-borne diseases, and safer, cleaner public spaces. As residents experience these tangible health and environmental benefits—and enjoy better access to amenities like organised waste-collection points, clean water outlets, and communal areas for food preparation and physical activity—their trust and pride in local initiatives deepen. Participants directly described the links between improved waste management, cleaner environments and better health, interpreted these as the reinforcing structure R1.
- Reinforcing loops R2 and R3 (Municipal support, waste management, and political gains) (Figures 4 and 5). The two loops together describe a cycle in which strengthened municipal and organisational actions for better waste management—including rigorous enforcement of urban planning and enhanced community and youth sensitisation on environmental risks and adaptation strategies. This mobilisation leads to the creation and upkeep of embankments and drainage systems, which in turn improve the efficiency of waste disposal and recycling practices, reduce drainage blockages, and elevate neighbourhood health and safety. As these improvements become visible, public perception of political gains will grow, reinforcing elected officials’ commitment to sustaining and expanding support for these initiatives. The positive feedback encourages municipal actors to allocate greater budgets and personnel to waste management, outreach campaigns, and infrastructure maintenance, which deepens trust between residents and local government and fuels further community participation in clean-up drives and advocacy. These loops were constructed by the authors by combining participants’ descriptions of how municipal support shapes waste management and infrastructure, and separate accounts of how visible improvements feed back into political gains and renewed support.
- Reinforcing loop R4 (Municipal Support, Informal Settlements, and Population Relocation. and Economic Activities) (Figure 6) illustrates a cycle in which increased municipal and organisational actions and support, paired with more rigorous enforcement of planning and management rules, work to curb the growth of informal settlements by facilitating formal development pathways (e.g., land titling, sanitation infrastructure, and community engagement). As informal settlements shrink, fewer households are forced to relocate out of flood-prone areas, which in turn diminishes the visibility of forced relocation programs and increases politicians’ perceived successes in “solving” the flooding crisis. Political gains decline when large-scale forced relocations occur, and the incentive for continued high-profile interventions weakens, eventually tempering further municipal support for flood resilience initiatives. Participants offered differing views on how municipal support, informal settlements and relocation interact; R4 therefore represents an interpretive synthesis that brings these views together as a single reinforcing loop rather than a feedback structure that participants explicitly named.

## Discussion

This study identified 14 critical variables that, in community actors’ accounts, shape urban resilience to flooding. These were organised into seven strategies (including waste management, tree planting, and community sensitisation), three drivers (such as municipal support and informal settlements), and four key outcomes (like improved hygiene and access to food). The analysis uncovered five feedback loops, four reinforcing and one balancing, highlighting how effective waste disposal, political engagement, and community mobilisation can either amplify or hinder resilience efforts. Participants described how municipal actions and local initiatives strengthened infrastructure and environmental management in specific neighbourhoods, while also pointing to persistent structural barriers such as weak planning enforcement, vulnerabilities in informal settlements, and politically driven interventions. Taken together, these empirical findings suggest that community-led strategies are central to responding to floods, but that their effectiveness is constrained by broader governance and equity issues.

The study used a Community-Based System Dynamics approach to identify these community resilience strategies to flooding, their related underlying drivers and health outcomes, as well as the emerging system feedback mechanisms in the city of Yaoundé. Our data show how the strategies identified by participants intersect with key determinants of health, including hygiene, access to food and opportunities for physical activity, rather than with NCD outcomes themselves. By mapping the relationships that participants described between waste management, drainage, hygiene, food production and public spaces, the CLD highlights plausible pathways through which flood resilience practices may shape NCD-related risk factors in cities of the Global South. We do not quantify NCD outcomes in this study, and these pathways should be interpreted as hypotheses for future testing rather than as empirically demonstrated effects.

The participatory CBSD methodology provided detailed insights into systemic interactions often overlooked by conventional flood management approaches. Reinforcing loops like community sensitisation, promoting better waste management, highlighting sustainability in community-led initiatives, and supporting the scalability of local actions (23). Conversely, balancing loops underscores the complexities and trade-offs in managing urban growth and disaster resilience, reflecting broader socio-economic tensions highlighted in the literature (24). These findings align with existing research emphasising the importance of integrated approaches for enhancing community resilience through local knowledge mobilisation, empowerment, and intersectoral collaboration (11, 25). At the same time, some of the broader interpretations we offer about NCD prevention and urban governance go beyond what can be directly observed in our data and should be understood as theoretically grounded propositions that build on, rather than strictly follow from, the CLD.

### Validity, contextual reflections and limitations

A major strength of this study is its participatory CBSD approach, which enables the handling of complex, non-linear, and dynamic problems like urban health and climate resilience, while ensuring community relevance, ownership, and actionable insights. The process builds local capacity in systems thinking, which can be sustained beyond the research project, benefiting both the local communities and the researchers. Thus, the process facilitated comprehensive dialogue and revealed nuanced local dynamics typically inaccessible via conventional methodologies (26). The resulting CLD should therefore be understood as a co-produced representation of how this particular group of actors in Yaoundé make sense of flood resilience, rather than as a definitive map of the wider urban system, and can be updated or adapted in future work or in other cities. The iterative validation process integral to CBSD enhances the study’s reliability by engaging stakeholders and refining/validating the model. In our case, validation took the form of a participatory review of the draft CLD during the workshop, followed by a team-based consistency check to confirm that all retained variables and links were grounded in at least one transcript or mental model. This process provides face and content validity for the model within this group of participants.

Context-specificity and transferability also warrant careful consideration. Yaoundé’s unique socio-political environment, characterised by rapid urbanisation, infrastructural deficits, and governance constraints, further complicates the generalisability of results. The city’s rapid growth brings dynamic changes in environmental risks and community vulnerabilities, while infrastructural challenges such as inadequate drainage and informal settlements influence both the problem and feasible solutions. Additionally, socio-cultural factors—such as community trust, local leadership structures, and cultural norms—play a critical role in shaping stakeholder engagement and resilience strategies. Comparatively, Awah et al. (11) identified similar vulnerabilities and community resilience mechanisms in Limbe, Cameroon, reinforcing the utility of participatory modelling for local flood management despite differing local dynamics. Morais et al. (16) further highlighted the adaptability of participatory systems approaches across diverse urban settings, including Belo Horizonte, Brazil, while emphasising that variations in institutional frameworks and socio-cultural contexts substantially influence outcomes and stakeholder engagement. These findings suggest that while participatory modelling holds promise across varied contexts, careful attention to local institutional capacities, socio-political nuances, and stakeholder heterogeneity is essential for effective adaptation and scaling. Taken together, these cross-site comparisons offer a partial form of triangulation for our findings: the close overlaps in key vulnerabilities, strategies, and institutional challenges increase confidence that the main variables and pathways represented in our CLD are not idiosyncratic to this sample, even though the specific feedback structures remain context-dependent.

Limitations in representation, inclusiveness and methodology also affect how our findings should be interpreted. The relatively small number of stakeholders involved, although sufficient to reach thematic saturation for the questions we explored, may have excluded some critical viewpoints from marginalised groups. In particular, residents with limited formal representation, such as people living in informal settlements, young people and people living with disabilities, were under-represented in our sample, so the CLD may understate barriers linked to insecure housing, accessibility, care responsibilities and other constraints that shape how these groups experience and respond to flooding. Certain assumptions—such as homogeneous stakeholder participation and institutional stability—may oversimplify the complex realities of Yaoundé’s context and limit the inclusiveness of the participatory process. Additionally, the qualitative nature of CBSD does not aim to generate statistically tested causal interpretations, suggesting that complementary quantitative studies would contribute to deeper validation and generalisation (27). In particular, future mixed-methods work could help to examine whether the feedback loops and pathways suggested by our CLD are observed in other neighbourhoods and cities, and to quantify their implications for NCD risk and health equity. More formal triangulation, for example, combining qualitative CLDs with quantitative flood, health or socio-economic data, was beyond the scope of this study but will be important for testing which of the hypothesised pathways and feedbacks are most strongly supported across different data sources.

Finally, the feasibility and scalability of the process itself depend on resources. This work was developed within the framework of a funded project, which provided the time and resources necessary to ensure its feasibility. While this may present challenges in terms of scalability and transferability to different local contexts, several aspects of the process suggest its adaptability. Most meetings were conducted online, in-person sessions leveraged the existing infrastructure of the local research team, and the modelling software used was available in free versions or could even be replicated using pen and paper when internet access was limited. These features indicate that CBSD methods can be flexibly adapted to suit the specific conditions and resources of diverse settings.

### Implications for policy and practice

Drawing on these empirical findings, we outline plausible implications for urban policy, disaster risk governance, and public health. These implications are grounded in the CLD, particularly waste management and hygiene (B1, R1), municipal support and political gains (R2, R3), and informal settlements and relocation (R4), as well as participants’ accounts. They extend beyond what this single qualitative case can demonstrate and should be interpreted as propositions rather than empirically tested effects. Acknowledging the climate change-NCD syndemic underscores the necessity for integrated resilience strategies that concurrently address environmental and public health outcomes (28).

Policy frameworks should actively incorporate community-led initiatives, harnessing local knowledge and capacity in formal disaster management strategies (29). Enhanced disaster risk governance, prioritising transparency, accountability, and community participation, is crucial for sustainable resilience-building, especially in Global South urban settings like Yaoundé (30). Our data indicate that inconsistent municipal support and politically motivated decision-making can disrupt or dilute community-led efforts, suggesting that governance reforms may be as important as technical investments in infrastructure.

Public health systems could benefit substantially from incorporating strategies identified by communities, such as infrastructure improvement and waste management, to mitigate disease risks associated with flooding (31). Participants in this study linked improved drainage, neighbourhood hygiene and access to food and public spaces to perceived health benefits, aligning urban planning with public health objectives, as advocated by the Lancet Commission on Planetary Health, further reinforces the critical need for holistic resilience strategies (32). For local communities, these partnerships contribute to strengthened local strategies, amplification, and resources.

### Recommendations

Based on these findings, we propose the following actionable recommendations to strengthen flood resilience at the local and city levels, specifying key roles for communities, policymakers, and other stakeholders:

- *Institutionalise community participation*. Policymakers and municipal authorities should formalise and sustain inclusive mechanisms for community engagement in disaster risk reduction and urban planning. Communities can organise neighbourhood committees, contribute local knowledge, and actively participate in decision-making forums, ensuring that resilience strategies are grounded in lived experience and that there is strong local ownership. Strengthening these participatory structures directly addresses the governance-related dynamics captured in loops R2–R4, where inconsistent municipal support and uneven responsiveness to communities risk weakening otherwise positive resilience feedback.
- *Invest in proven local solutions*. Government agencies and donor organisations should allocate targeted resources to expand adequate, community-identified infrastructure and environmental management practices, such as improved drainage, green buffers, and flood-resistant housing. Community groups can lead on maintenance, help monitor effectiveness, and encourage the uptake of these locally appropriate interventions. Our data show that such actions are central to how residents already manage flood risk in Yaoundé. These investments act on the waste, drainage and hygiene dynamics summarised in loops B1 and R1, where sustained infrastructure and environmental management can shift the system away from recurrent flooding and poor hygiene towards reinforcing improvements in neighbourhood conditions.
- *Foster intersectoral collaboration*. National and municipal agencies should strengthen coordination between the health, urban planning, and environmental sectors to address the intersecting risks of flooding and NCDs. This includes joint planning, shared data systems, and integrated implementation frameworks. Civil society can play an advocacy role and help monitor health and environmental outcomes. The feedback structures in the CLD suggest that uncoordinated actions may inadvertently shift problems from one sector to another rather than resolving them. In particular, loops R2–R3 show how fragmented action on waste management, planning enforcement, and health promotion can undermine potential reinforcing benefits, whereas coordinated efforts could help to stabilise and amplify positive trajectories.
- *Promote climate and health literacy*. Educators, local authorities, and media should integrate environmental education, flood awareness, and climate resilience campaigns into school curricula, community programs, and public messaging. Community leaders and youth groups can facilitate local workshops and peer learning to encourage adaptive behaviours and preparedness. Participants in our study repeatedly highlighted sensitisation as a key resilience strategy. This recommendation targets the social and behavioural components of loops R1–R3, where community sensitisation was seen as essential for sustaining waste management practices, hygiene behaviours and engagement with municipal initiatives.
- *Advance mixed-methods evidence for policy*. Researchers and policymakers should complement qualitative participatory modelling with quantitative approaches, such as scenario modelling and cost-effectiveness analysis, to assess the long-term impact of community-led interventions. Communities can contribute to data collection and evaluation, helping to strengthen the evidence base for adaptive planning. Such work will be critical for testing which of the hypothesised feedback loops (B1, R1–R4) are most influential in practice and for quantifying the potential impact of intervening at the leverage points identified in the CLD.

## Conclusion

This study highlights the complex interaction between community resilience strategies to floods and the strategy drivers and outcomes in Yaoundé, Cameroon. It underscores the value of participatory systems-mapping approaches in elucidating community resilience strategies to urban flooding. Community-Based System Dynamics methodologies provide robust tools for capturing complex urban dynamics and identifying strategic intervention points to foster resilience. Our CLD proposes hypotheses about how these strategies may shape NCD-related determinants, which future quantitative and mixed-methods studies should test. As climate risks intensify, participatory methods will be increasingly vital for developing sustainable, health-enhancing resilience in rapidly urbanising Global South contexts.

## Data Availability

The original contributions presented in the study are included in the article/supplementary material, further inquiries can be directed to the corresponding author/s.
